# An interview with Roberto Lima Filho

**DOI:** 10.1590/2177-6709.21.3.030-038.int

**Published:** 2016

**Authors:** O. H. "Chip" Rigsbee III, Allen H. Moffitt, James Vaden, Andrew J. Haas, Eustaquio Araujo

**Affiliations:** » DDS, Indiana University, School of Dentistry, Bloomington, IN, USA. » MSc, Certificate in Orthodontics, University of Illinois, School of Dentistry, Bloomington, IN, USA. » Part-time faculty, Indiana University, School of Dentistry, Department of Orofacial Development, Bloomington, IN, USA . » Peer Reviewer, American Journal of Orthodontics and Dentofacial Orthopedics. » Member of the Angle Midwest Component of the Edward H. Angle Society of Orthodontists. » Admissions Committee Chairman, Angle Midwest, Edward H. Angle Society of Orthodontists.; » DDS (Magna cum laude), University of Kentucky, School of Dentistry, Lexington, KY, USA. » MSc in Dentistry, Certificate in Orthodontics, University of Washington, School of Dentistry, Seattle, WA, USA. » Clinical Assistant Professor, University of Tennessee, Knoxville, TN, USA. » Assistant Clinical Professor, Vanderbilt University, Division of Orthodontics, Nashville, TN, USA. » Associate Editor of Continuing Education for the American Journal of Orthodontics and Dentofacial Orthopedics. » Former Director of the American Board of Orthodontics. » Member of the Angle Midwest Component of the Edward H. Angle Society of Orthodontists. » Fellow of the American College of Dentists, Fellow of the International College of Dentists, and College of Diplomates of ABO.; » DDS, University of Tennessee, School of Dentistry, Knoxville, TN, USA. » MSc, Certificate in Orthodontics, University of Tennessee, School of Dentistry, Knoxville, TN, USA. » Commissioner for Orthodontics for the Commission on Dental Accreditation (CODA). » Professor and Former Chairman of Orthodontics, University of Tennessee, Knoxville, TN, USA. » Adjunct Clinical Professor, University of Michigan, Department of Orthodontics, Ann Arbor, MI, USA. » Director of the Charles Tweed Foundation. » Past President of the American Board of Orthodontics. » Member of the Angle Midwest Component of the Edward H. Angle Society of Orthodontists. » Fellow of the American College of Dentists, Fellow of the International College of Dentists, and College of Diplomates of ABO.; » DDS and MSc, University of Illinois, Champaign, IL, USA. » Professorship, University of Illinois, Loyola University of Chicago, and The Ohio State University. » Continuing education appointments: Temple University, Tufts University, Pittsburg University, Indiana University and the US Army Orthodontics Program. » Vast contribution to research and clinics. First to introduce all three forms of palatal expansion: rapid, semi-rapid, and slow via Kloehn face bow manipulation. » First to employ surgically assisted rapid palatal expansion (corticotomy).; » Dental surgeon, Universidade Federal de Minas Gerais (UFMG), Belo Horizonte, Minas Gerais, Brazil. » Certificate and MDS, University of Pittsburgh, Pittsburgh, PA, USA. » Pete Sotiropoulos Professor of Orthodontics, Associate Director and Graduate Orthodontics Clinic Director at the Center for Advanced Dental Education, Saint Louis University. » Recipient of the Louise Ada Jarabak Award of the American Association of Orthodontists Foundation (AAOF). » Author of the book » Recognizing and Correcting Developing Malocclusions." » Member of the Angle Society of Orthodontics, Midwest Component. » Diplomate of the American and Brazilian Board of Orthodontics and Dentofacial Orthopedics (BBO). » Past President of the Brazilian Board of Orthodontics and Dentofacial Orthopedics (BBO). » Former President of Pontifícia Universidade Católica de Minas Gerais (PUC-MG).

» Post-Graduate in Orthodontics, University of Illinois, Chicago, IL, USA (1975).

» PhD, Universidade Federal do Rio de Janeiro (UFRJ), Rio de Janeiro/RJ Brazil (2006). 

» First Brazilian to become a Diplomate of the American Board of Orthodontics (ABO) (1992).

» Member of the Edward H. Angle Society of Orthodontists (Midwest Component) since 2002. 

» Founding President of the Brazilian Board of Orthodontics and Dentofacial Orthopedics (BBO). 

» Past-President of the Edward H. Angle Society of Orthodontists (Midwest Component) (2015-2016). 

» Author of the textbook "*Ortodontia: Arte e Ciência.*" 

» Honor for 12 years of service on the Board of Directors, Illinois Orthodontic Alumni Association (1992-2004). 

» CDABO Case Report of the Year published in the American Journal of Orthodontics & Dentofacial Orthopedics (2003). 

**Figure:**
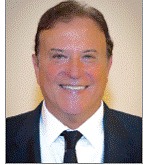

Quando fui convidado para coordenar uma entrevista com Roberto Lima, me senti extremamente honrado. O que posso dizer sobre o Roberto? Um homem com um currículo impressionante e que contribuiu muito para a Ortodontia no Brasil e nos Estados Unidos? Sem dúvida, tentar destacar os feitos de sua carreira é uma tarefa difícil. Seu currículo fala por si mesmo. Sendo assim, em vez de apresentar o colega, líder e ortodontista de destaque que ele é, aproveitarei a oportunidade para apresentar o homem, seu caráter, carisma e seus valores. O pai de Roberto foi dentista e pós-graduou-se em Dentística pela *University of Washington*, Seattle, EUA, o que praticamente abriu as portas para Roberto seguir os passos dele. Roberto graduou-se em Odontologia pela Pontifícia Universidade Católica de Campinas (PUC-Campinas), São Paulo, Brasil, e em Ortodontia pela *University of Illinois*, Chicago, IL, EUA. No entanto, Roberto não estava sozinho, em Campinas ele conheceu e se casou com Baby, seu grande amor e sua grande paixão. Juntos, eles se formaram em Odontologia e tornaram-se o primeiro casal a ingressar no programa de pós-graduação da *University of Illinois* - seguindo o legado do pai de Roberto e, também, de seu tio, ex-aluno da *University of Illinois*. Tiveram dois filhos, Roberto Neto, advogado, e Carolina, ortodontista. Roberto não conta mais com a presença de Baby junto a ele, porém, encontra amor e parceria em seus filhos e quatro netos. Além da Ortodontia, Roberto ama fotografia e esportes, como tênis, bicicleta e caminhadas de longa distância. É uma das poucas pessoas que conheço que completou o Caminho de Santiago de Compostela - uma conquista e tanto! No livro "Do chão ao coração: *uma história do Caminho*", que acaba de ser lançado, ele compartilha com todos a sua experiência que, sem dúvida, deve ter sido incrível. Como podemos ver, há muito mais a se dizer sobre Roberto, um profissional dedicado e um exemplo a ser seguido pelas novas gerações. Tenho orgulho de ser seu amigo e colega de profissão. Obrigado, Roberto, por trazer liderança e carisma para a nossa profissão. Obrigado pelo seu exemplo de dedicação e profissionalismo. Também agradeço aos Drs. O. H. "Chip" Rigsbee III, Allen Moffitt, James Vaden, e ao nosso querido Andy Haas, pelas contribuições dadas a esta entrevista.

Eustaquio Araujo - coordenador da entrevista 

When I was invited to coordinate Roberto Lima's interview, I felt extremely honored. What can I say about Roberto? A man with an impressive curriculum and who has done so much for Orthodontics in Brazil as well as in the United States of America? Definitely it becomes a hard task to try to pinpoint the highlights of his career. His *résumé* speaks for itself. Therefore, instead of introducing the colleague, leader and outstanding orthodontist he is, I will use this opportunity to introduce the man*,* his character, charisma and values. Roberto's father was a dentist and obtained his graduate degree in Operative Dentistry from the University of Washington, Seattle, USA. That basically opened the doors for Roberto to follow the footsteps of his father. He graduated in Dentistry from Pontifícia Universidade Católica de Campinas (PUC-Campinas), São Paulo, Brazil, and in Orthodontics at the University of Illinois, Chicago, IL, USA. However, Roberto was not alone: in Campinas, he met and married Baby, his great love and passion. Together they graduated in Dentistry and were the first husband/wife team to enter the same graduate program at the University of Illinois. They followed the legacy of Roberto's father, alumnus from University of Washington, and grandfather, an alumnus from the University of Illinois. They had two children, Roberto Neto and Carolina, a lawyer and an orthodontist, respectively. Roberto no longer has the physical presence of Baby with him; however, he finds love and partnership in his children and four grandchildren. In addition to Orthodontics, Roberto loves photography, plays tennis, practices cycling and loves to take long walks. He is one of the few people I know who have walked *Camino de Santiago de Compostela* until the end. What an accomplishment! In the book, "*Do Chão ao Coração: Uma história do Caminho,*" which has just come out, he shares with everyone his experience that certainly must have been incredible. As you can see, there is much more I can say about Roberto, a devoted professional and a role model for generations to come. I am so proud to be one of his friends and colleagues. Thank you, Roberto, for bringing your leadership and charisma to our profession. Thank you for your example of dedication and professionalism. I also would like to thank Drs. O. H. "Chip" Rigsbee III, Allen Moffitt, James Vaden, and our beloved Andy Haas for their contributions to this interview.

Eustaquio Araujo - interview coordinator 

This interview will address Roberto Lima's experience with the Angle Society, the Brazilian Board of Orthodontics and Dentofacial Orthopedics (BBO), Clinical Orthodontics and our specialty. The questions were grouped by subject and we thank Dr. Lima for his answers. So, let's enjoy it!

## 1. How has the Angle Society influenced your professional career? Allen H. Moffitt

I apply the knowledge and experience I have gained within the Society in the day-to-day treatment of my patients all. I have gained a lot and continue to do so, but my patients have also gained with an improved high-quality treatment. Being part of a group such as the Angle Society is representative and means that we are in contact with the best of what is available in the world, both clinically and scientifically. 

## 2. You are the immediate past President of the Angle Midwest Society of Orthodontists and have devoted many years of service to that organization. What was behind that decision? Chip Rigsbee

I had heard of the Angle Society for several years and I knew that it was one of the best groups in our specialty. I never imagined that one day I would become a member or even its President, especially being the first at-large President in the history of the Society. The process of becoming a member, although enjoyable, is not easy. It requires years of hard work and dedication. However, you are compensated by all the experience you gain; not only during the membership process, but also with continuous learning as a member. I have no doubts whatsoever that all those years serving as Chair of the Admissions Committee and then as President gave me back much more than I invested in the Society. This experience has been incredible.

## 3. In what ways are you involved with giving back to your community, whether in service-oriented or leadership roles? Chip Rigsbee

I tried to give back to my orthodontic community by founding the Brazilian Board of Orthodontics and Dentofacial Orthopedics (BBO) along with other Brazilian colleagues. The Board was founded in September 2002 to fill a gap in the Brazilian system which at that time did not stimulate or control the quality of orthodontic treatment. The examinations provide a unique opportunity for candidates to review their practice, reflect on the importance of carefully maintaining quality records, evaluate mechanical control in performing treatment and pay attention to final details. I also share my knowledge and experience through lectures and round tables both in my country and abroad.

## 4. You were deeply involved with the set up of BBO. What or who encouraged you to help setting up a certifying board in Brazil? Was this experience a good one and has it raised the level of Orthodontics in Brazil? James Vaden

**Figure 1:**
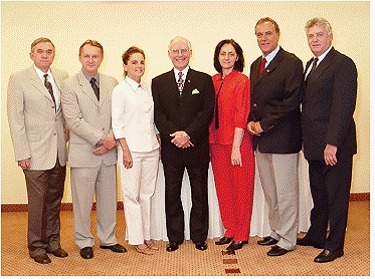
Board of Directors at the first examination of the Brazilian Board of Orthodontics and Dentofacial Orthopedics with the special participation of Jack Dale. From left to right: Carlos Vogel, Nelson Mucha, Telma Araujo, Jack Dale, Ana Maria Bolognese, Roberto Lima and Estelio Zen.

I was encouraged by my own experience with ABO certification in 1992. The idea of setting up the Brazilian Board of Orthodontics and Dentofacial Orthopedics (BBO) came up from the need to promote the achievement of clinical excellence standards in the orthodontic practice in my country. In 1998, the Brazilian Association of Orthodontics and Dentofacial Orthopedics elected a Special Committee to constitute the first Board of Directors. Along with my colleagues, we conducted the first examination in 2004 with the special participation of Jack Dale, renowned Canadian orthodontist and former ABO President (Fig 1). The experience with BBO has been excellent, as the awareness of professional qualification relevance has expanded. The Brazilian Board of Orthodontics and Dentofacial Orthopedics (BBO) has become synonymous with qualification and adequate training to perform successful treatment.

## 5. What philanthropic projects with which you have been involved have provided you with the greatest satisfaction and why? Allen H. Moffitt

Currently I am leading a social movement in my area, a spontaneous group with cross-party ideals, struggling for a country where every citizen can be active in setting up a prosperous and dignified society. We operate both in the community where I live as well as nationally. On another note, last year there was an outbreak of dengue fever in my native town. The group acted by raising the population's awareness and implementing effective measures against it. As a result, in 2016, our city has one of the lowest rates of the disease in the entire country.

## 6. You have been exposed to at least two different orthodontic realities/cultures: the American and Brazilian ones. How do you compare Orthodontics in Brazil and in the USA? Eustaquio Araujo

Brazilian Dentistry is strongly influenced by the American culture. The quality of the best orthodontists in Brazil is comparable to those in the USA. However, in the United States, the majority of the population has economic conditions that allow them to have access to leading-edge technology. In Brazil, only a few can afford and demand the most sophisticated treatment available. The majority of patients is forced to accept services on a much more limited budget.

## 7. In your orthodontic career, which technique or procedure has had the biggest impact on your orthodontic therapy? Allen H. Moffitt

**Figure 2:**
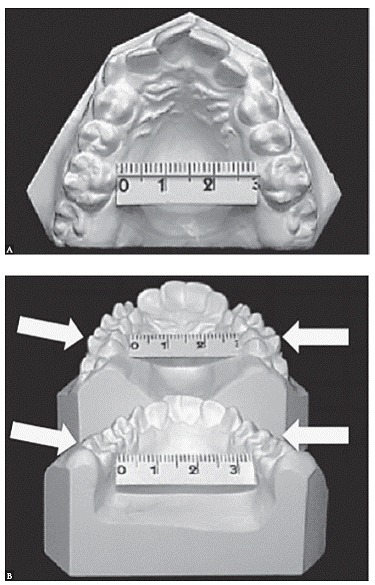
Diagnosing transverse discrepancy: pretreatment casts of a 12-year-old patient. Note the maxillary deficiency and unfavorable inclination of posterior teeth (maxillary teeth inclined buccally and mandibular teeth inclined lingually).

**Figure 3:**
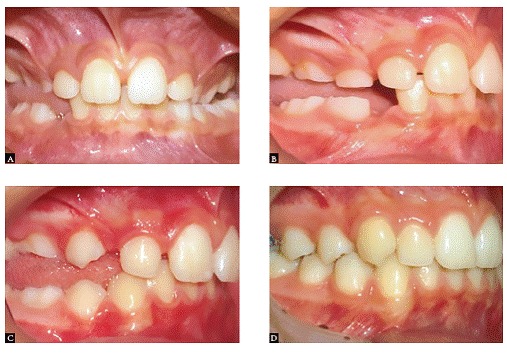
A) An example of a case nearly finished with minimal interference: A) Class I transverse skeletal maxillary deficiency and posterior open bite. B) Rapid palatal expansion to correct transverse deficiency and gain space for maxillary canine. C) Cervical headgear to gain additional space and D) at the end of the first phase of treatment with no fixed appliances at that time.

**Figure 4:**
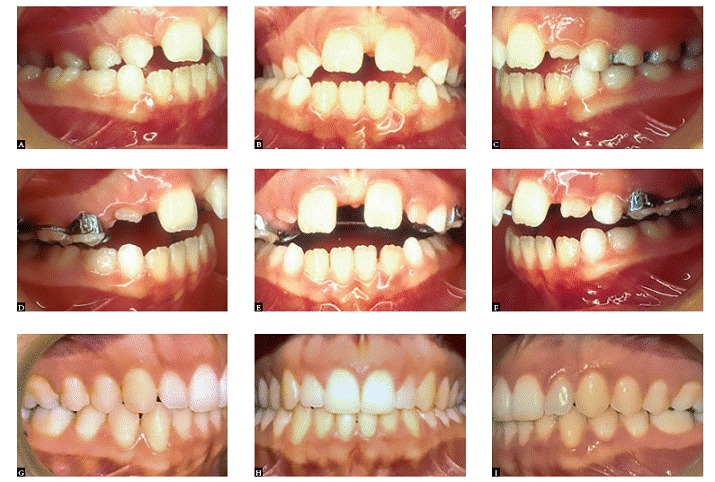
Intraoral photographs: right lateral, central, and left lateral views at pretreatment (A, B and C), post expansion (D, E and F), and post-treatment (G, H and I). The only treatment provided was the rapid palatal expansion (RPE). After expansion, the mandible was carried forward to its normal position, resulting in spontaneous correction of Class II malocclusion on the right side.

**Figure 5:**
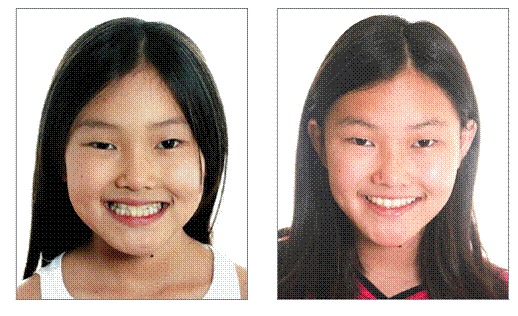
Skeletal Class III patient after phase I treatment. Note the improvement in patient's smile and the alignment of teeth after the orthopedic phase. No fixed or removable appliances were used for alignment at this phase. The increase in the amount of maxillary incisors seen contributed tremendously to improve the esthetics of patient's smile.

The procedure exerting the biggest impact on orthodontic therapy is precise diagnosis (Fig 2). This is the key to success in orthodontic treatment. An improper diagnosis, which is followed by incorrect treatment, is also the number one cause of relapse. Over my orthodontic career, I have always committed to treatment concepts that fit patients' needs, not the other way around, while using techniques that allow me to accomplish the most with the least intervention (Fig 3). I agree with Dr. Riedel when he says: "I have always been most proud of those patients for whom I have been able to apply a minimum of mechanical aids to achieve ultimate success in a beautiful dentition derived from my guidance of natural forces with little or no mechanical intervention" (Figs 4, 5).

## 8. You have traveled the world and have met many orthodontists. Who are the two or three who have influenced you the most and why have they done so? James Vaden

This is a difficult question because there have been so many orthodontists who have helped me immensely; outstanding professionals for whom I have the deepest consideration and respect. If I have to single out one or two, I must mention Andy Haas and Jim Jensen. Andy Haas, a professor of mine at University of Illinois, introduced me to the principles of early treatment and Dentofacial Orthopedics. Jim Jensen was my sponsor to the Angle Society. I had the privilege to spend time in his office for a couple of weeks and because of him I was able to improve my abilities in dealing with extraction treatment, in addition to finishing my cases with excellence. 

## 9. In rapid palatal expansion (RPE) cases in which there is a delay of six months or more before initiating orthodontic treatment, have you observed that mandibular buccal teeth expand? Andrew J. Haas

**Figure 6:**
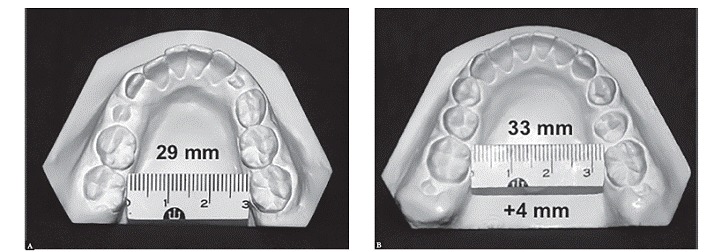
Casts of a 10-year-old patient (mandibular occlusal view) at pretreatment (A) and progress (two years later) (B). Note the increase in intermolar width just with RPE. No fixed or removable appliances were used in the lower arch in this treatment phase.

Yes, I have. My daughter Carolina studied the spontaneous changes in mandibular dentition under the influence of maxillary expansion. Her sample consisted of 30 consecutive patients who were followed in the long term without any additional treatment. She concluded that mandibular intermolar arch width increased significantly. The long-term outcomes showed a remarkable and positive clinical stability in mandibular arch width dimensions in patients treated only by means of RPE (Fig 6). 

## 10. Why do you think this happens? Andrew J. Haas

I actually agree with your studies and statement disclosed in 1961: "When the maxilla is expanded 12-14 mm, noticeable spontaneous expansion will occur in the lower dental arch due to altered muscle balance between the tongue and buccinator muscles, as they affect the lower dental arch. That is, a permanent increase in maxillary apical base which leads to a spontaneous, permanent and significant increases in mandibular arch width." Now we have a better understanding that the position of the lower dentition may be more significantly influenced by maxillary skeletal morphology than by the size and shape of the mandible.

## 11. Do you think that in non-orthopedic expansion the fact that the forces of occlusion and muscle balance are not favorably altered (as in orthopedic expansion) might be the main cause of relapse? Andrew J. Haas

**Figure 7:**
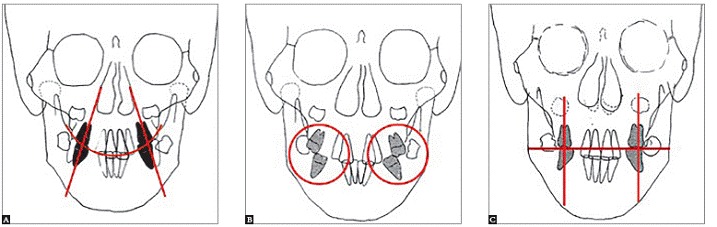
Skeletal Class II, Division 1 malocclusion patient during mixed dentition: Posteroanterior tracings. A) Pretreatment. Note unfavorable axial inclination of posterior teeth. Lingual cusps of maxillary molars are inferior to buccal cusps. B) Post expansion. Note overexpansion of maxillary posterior teeth. C) Post-treatment. Note unfavorable axial inclination of maxillary posterior teeth has been corrected.

Ignoring transverse skeletal discrepancies and just treating these abnormalities by dental expansion will result in improper buccolingual inclination of posterior teeth, thereby leading to unstable results. The only stability we can expect is at the skeletal level, which results in favorably altered muscle balance with the buccal teeth uprighted over their maxillary bony bases (Fig 7).

## 12. Is there a way to make patients compliant with extra- and intraoral appliances as well as oral hygiene nowadays? Has that variable changed the orthodontic decisions you made in the past and the ones you make today? Eustaquio Araujo

**Figure 8:**
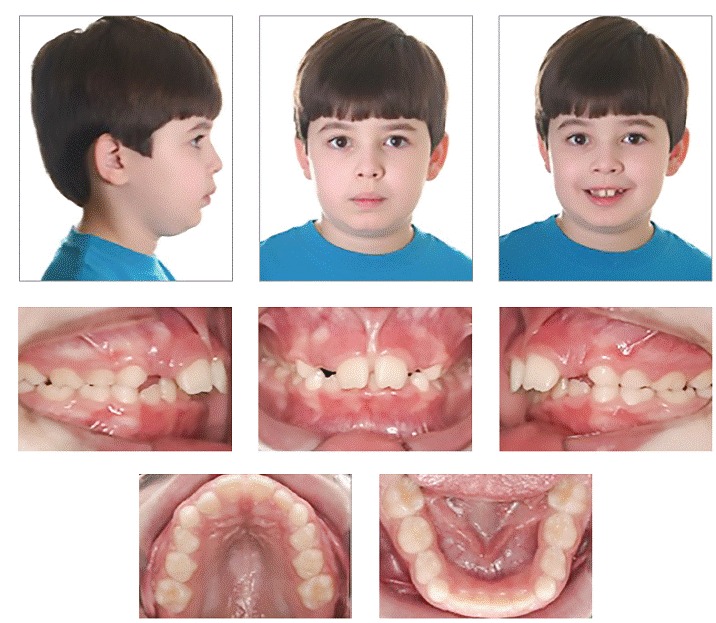
Typical Class II, Division 1 patient with transverse maxillary deficiency.

**Figure 9:**
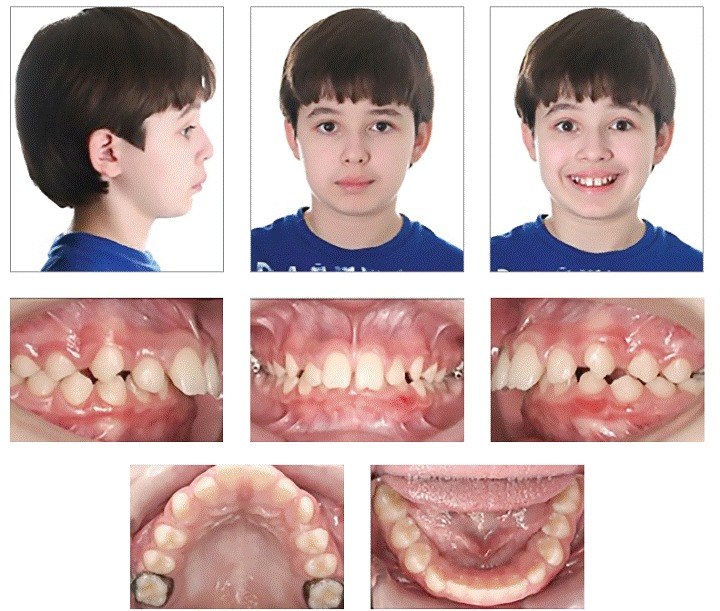
Note Class II molar correction with transseptal fibers producing significant improvement in the buccal segment with bicuspids assuming a solid Class I occlusion.

After many years of experience, I have seen that the relationship between patient and orthodontist has changed significantly regarding compliance. In general, particularly in larger cities, colleagues have reported that compliance has diminished substantially. However, in my practice, I still have a relatively good cooperation. I have used the same strategy since I started in my practice, which is to communicate with the patient about the importance of following treatment advice. My treatment plan consultation takes at least one hour. It is the appointment during which I present options and educate both parents and child. I find that it is more important for the child to understand what is going to happen in respect to his/her treatment, increasing the chances for greater involvement. Thus, dentist and patient together strive to achieve the same treatment outcomes (Figs 8, 9).

## 13. What is the future of Orthodontics in the next 25 years? What can be done to preserve it as a specialty, since there are many factors that seem to be impacting it in every part of the world? James Vaden

Over the last few years, we have seen a tendency towards greater technological involvement, such as computerized bracket placement devices and computer-aided manufactured acrylic appliances, to improve efficiency and consequently make our profession a better product to sell. To my view, this tendency will not replace the orthodontist's ability; it will only add quality and efficiency to treatment. However, we cannot forget that each patient has a different type of malocclusion and treatment must be adapted to individual needs; quality results take time and attention to detail. Researches on malocclusion and on the psychological impact of facial change on personality development using more biological bases are much more relevant than prescription treatment dictated by business. As Mark Hans stated: "In the future, we will have more time to think and we need to think about the art and science of Orthodontics rather than the business of practice."

## 14. Could you explain the present craze of the orthodontic market in Brazil? Eustaquio Araujo

Over the last few years, there has been an extremely high number of private universities of poor quality opening in Brazil. This has resulted in large numbers of badly qualified professionals entering the work market without hopes of survival. Education cannot be seen as a business only. The focus on profit most likely conflicts with quality training of students and with the goal of making them capable professionals. Many professionals incorrectly see in Orthodontics an opportunity to become rich. However, our specialty is much more than that and cannot be summarized as the practice of bracket placement only; our specialty is based on art and science. We need to raise the standards of education in Brazil, an enormous, but not impossible task that needs to be started immediately.

## 15. What has given you the greatest satisfaction in your profession? Chip Rigsbee

This is a great question to bring this interview to a close. The answer is easy: the smiles that appears on patients' faces as an expression of happiness and gratitude for treatment results. It gives me a feeling of achievement and satisfaction; a magnificent reward that the profession has given me my entire professional life.

## O. H. "Chip" Rigsbee III 

» DDS, Indiana University, School of Dentistry, Bloomington, IN, USA.

» MSc, Certificate in Orthodontics, University of Illinois, School of Dentistry, Bloomington, IN, USA.

» Part-time faculty, Indiana University, School of Dentistry, Department of Orofacial Development, Bloomington, IN, USA .

» Peer Reviewer, American Journal of Orthodontics and Dentofacial Orthopedics.

» Member of the Angle Midwest Component of the Edward H. Angle Society of Orthodontists.

» Admissions Committee Chairman, Angle Midwest, Edward H. Angle Society of Orthodontists.

## Allen H. Moffitt

» DDS (*Magna cum laude)*, University of Kentucky, School of Dentistry, Lexington, KY, USA.

» MSc in Dentistry, Certificate in Orthodontics, University of Washington, School of Dentistry, Seattle, WA, USA.

» Clinical Assistant Professor, University of Tennessee, Knoxville, TN, USA.

» Assistant Clinical Professor, Vanderbilt University, Division of Orthodontics, Nashville, TN, USA. 

» Associate Editor of Continuing Education for the American Journal of Orthodontics and Dentofacial Orthopedics.

» Former Director of the American Board of Orthodontics.

» Member of the Angle Midwest Component of the Edward H. Angle Society of Orthodontists.

» Fellow of the American College of Dentists, Fellow of the International College of Dentists, and College of Diplomates of ABO.

## James Vaden

» DDS, University of Tennessee, School of Dentistry, Knoxville, TN, USA.

» MSc, Certificate in Orthodontics, University of Tennessee, School of Dentistry, Knoxville, TN, USA.

» Commissioner for Orthodontics for the Commission on Dental Accreditation (CODA).

» Professor and Former Chairman of Orthodontics, University of Tennessee, Knoxville, TN, USA.

» Adjunct Clinical Professor, University of Michigan, Department of Orthodontics, Ann Arbor, MI, USA.

» Director of the Charles Tweed Foundation.

» Past President of the American Board of Orthodontics.

» Member of the Angle Midwest Component of the Edward H. Angle Society of Orthodontists.

» Fellow of the American College of Dentists, Fellow of the International College of Dentists, and College of Diplomates of ABO.

## Andrew J. Haas 

» DDS and MSc, University of Illinois, Champaign, IL, USA.

» Professorship, University of Illinois, Loyola University of Chicago, and The Ohio State University.

» Continuing education appointments: Temple University, Tufts University, Pittsburg University, Indiana University and the US Army Orthodontics Program.

» Vast contribution to research and clinics. First to introduce all three forms of palatal expansion: rapid, semi-rapid, and slow via Kloehn face bow manipulation.

» First to employ surgically assisted rapid palatal expansion (corticotomy). 

## Eustaquio Araujo

» Dental surgeon, Universidade Federal de Minas Gerais (UFMG), Belo Horizonte, Minas Gerais, Brazil.

» Certificate and MDS, University of Pittsburgh, Pittsburgh, PA, USA.

» Pete Sotiropoulos Professor of Orthodontics, Associate Director and Graduate Orthodontics Clinic Director at the Center for Advanced Dental Education, Saint Louis University.

» Recipient of the Louise Ada Jarabak Award of the American Association of Orthodontists Foundation (AAOF).

» Author of the book "Recognizing and Correcting Developing Malocclusions."

» Member of the Angle Society of Orthodontics, Midwest Component.

» Diplomate of the American and Brazilian Board of Orthodontics and Dentofacial Orthopedics (BBO).

» Past President of the Brazilian Board of Orthodontics and Dentofacial Orthopedics (BBO).

» Former President of Pontifícia Universidade Católica de Minas Gerais (PUC-MG).

